# Heterogeneity in Spatial Inequities in COVID-19 Vaccination across 16 Large US Cities

**DOI:** 10.1093/aje/kwac076

**Published:** 2022-04-22

**Authors:** Usama Bilal, Pricila H Mullachery, Alina Schnake-Mahl, Heather Rollins, Edwin McCulley, Jennifer Kolker, Sharrelle Barber, Ana V Diez Roux

**Affiliations:** Urban Health Collaborative, Drexel Dornsife School of Public Health, Philadelphia, PA; Department of Epidemiology and Biostatistics, Drexel Dornsife School of Public Health, Philadelphia, PA; Urban Health Collaborative, Drexel Dornsife School of Public Health, Philadelphia, PA; Urban Health Collaborative, Drexel Dornsife School of Public Health, Philadelphia, PA; Urban Health Collaborative, Drexel Dornsife School of Public Health, Philadelphia, PA; Urban Health Collaborative, Drexel Dornsife School of Public Health, Philadelphia, PA; Urban Health Collaborative, Drexel Dornsife School of Public Health, Philadelphia, PA; Department of Health Management and Policy, Drexel Dornsife School of Public Helath, Philadelphia, PA; Urban Health Collaborative, Drexel Dornsife School of Public Health, Philadelphia, PA; Department of Epidemiology and Biostatistics, Drexel Dornsife School of Public Health, Philadelphia, PA; Urban Health Collaborative, Drexel Dornsife School of Public Health, Philadelphia, PA; Department of Epidemiology and Biostatistics, Drexel Dornsife School of Public Health, Philadelphia, PA

**Keywords:** COVID-19, SARS-COV-2, urban health, health disparities, neighborhoods, health equity, vaccination

## Abstract

Differences in vaccination coverage can perpetuate COVID-19 disparities. We explored the association between neighborhood-level social vulnerability and COVID-19 vaccination coverage in 16 large US cities from the beginning of the vaccination campaign in December 2020 through September 2021. We calculated the proportion of fully vaccinated adults in 866 zip code tabulation areas (ZCTA) of 16 large US cities: Long Beach, Los Angeles, Oakland, San Diego, San Francisco, and San Jose (CA); Chicago (IL); Indianapolis (IN); Minneapolis (MN); New York City (NY); Philadelphia (PA); and Austin, Dallas, Fort Worth, Houston, and San Antonio (TX). We computed absolute and relative total and Social Vulnerability Index(SVI)-related inequities by city. COVID-19 vaccination coverage was 0.75 times (95% CI 0.69-0.81) or 16% (95% CI 12.1-20.2%) percentage points lower in neighborhoods with the highest social vulnerability as compared to those with the lowest. These inequities were heterogeneous, with cities in the West region generally displaying narrower inequities in both the absolute and relative scales. The SVI domains of socioeconomic status and household composition & disability showed the strongest associations with vaccination coverage. Inequities in COVID-19 vaccinations hamper efforts to achieve health equity, as they mirror and could lead to even wider inequities in other COVID-19 outcomes.

Through December 2021, the COVID-19 pandemic has taken the lives of over 800,000 people in the US. The burden of COVID-19 has been disproportionate among minoritized populations and persons of lower socioeconomic position. We previously reported higher positivity ratios, incidence and mortality rates in areas of high social vulnerability(1), and these patterns have been replicated in other settings worldwide(2-4).

In December 2020, the first two vaccines against SARS-CoV-2 infection were authorized by the FDA. A vaccination roll-out started nationwide, with different prioritization schedules by jurisdiction, but that generally focused first on older adults, healthcare workers, and long-term care facility residents and staff(5). By April 19th, 2021, all states had opened vaccine eligibility to all adults aged 16 or above. These vaccines have proven highly efficacious(6, 7) and effective(8), and represent one of the key tools to address the ongoing pandemic.

In the context of higher incidence and mortality rates among low socioeconomic status and minoritized populations, calls for prioritizing these populations emerged (9-11). Early

ORIGINAL UNEDITED MANUSCRIPT

recommendations on vaccine allocation proposed the targeting of neighborhoods with high social vulnerability(11). A modeling study reported that a strategy of geographic targeting of high-risk neighborhoods would result in lower overall COVID-19 mortality as compared to age-based strategies alone (9). However, vaccination policies targeting high-risk neighborhoods have not been widespread, and reports of inequities in vaccination across counties and population subgroups have quickly emerged (12-14).

Characterizing social and spatial inequities in cities is critical to developing appropriate interventions and policies to increase COVID-19 vaccination among underserved populations, helping control the pandemic, and reducing health disparities. This is especially important in large cities where social inequalities are more prevalent (15), and whose high-vulnerability neighborhoods tend to bear the highest overall COVID-19 mortality burden (9). Therefore, the aim of this study was to characterize spatial and social inequities in vaccination coverage in 16 large US cities and examine heterogeneities in spatial and social inequities across cities. We hypothesized that neighborhoods with higher levels of social vulnerability would have lower vaccination coverage, and that the magnitude of inequalities would vary widely by city.

**Methods**

Setting

We obtained data from the *BCHC COVID-19 Health Inequities in Cities Dashboard(16)*, which compiles data on COVID-19 inequities in the jurisdictions of health departments that are members of the Big Cities Health Coalition (BCHC). For this analysis, we used data on the total number of fully vaccinated individuals by neighborhood in six cities of California (Long Beach, Los Angeles, Oakland, San Diego, San Francisco, and San Jose), five cities of Texas (Austin, Dallas, Fort Worth, Houston, and San Antonio), Chicago (IL), Indianapolis (IN), Minneapolis

ORIGINAL UNEDITED MANUSCRIPT

(MN), New York City (NY) and Philadelphia (PA). Neighborhoods were defined as zip code tabulation area (ZCTAs from the 2010 census vintage) of residence. ZCTAs, while imperfect proxies for neighborhood, represent a practical way to collect data during a public health emergency. We selected ZCTAs that overlapped, at least partially, with the extent of each city (defined as a census place). Data was obtained cumulatively from onset of vaccination through the end of September 2021 (dates varying from September 22, 2021, to September 29, 2021). **Web Table 1** details the specific data sources for each city, also available in the online Dashboard (www.covid-inequities.info).

Outcomes

The main outcome was the proportion of the total population that was fully vaccinated in each neighborhood. Full vaccination was defined by health departments as individuals that received two doses of mRNA-based vaccines (Pfizer-BioNTech [New York, NY] or Moderna [Cambridge, MA] vaccines) or one dose of the Janssen (Beerse, Belgium) vaccine.

Predictors

The main neighborhood-level predictor examined was the 2018 CDC's Social Vulnerability Index (SVI)(17) at the neighborhood level, calculated using data from the 5-year 2015-2019 American Community Survey. The SVI reflects the community's ability to prevent human suffering and financial loss in the event of disaster, including disease outbreaks(17) and has been used as a predictor of COVID-19 related outcomes in prior work(1, 14). The SVI includes 15 variables in four domains: socioeconomic status, household composition & disability, minority status & language, and housing type & transportation, along with a summary score for all four domains. Neighborhoods in each city were ranked according to the values of 15 variables in each domain, and national percentile ranks were computed for each neighborhood by domain and for

ORIGINAL UNEDITED MANUSCRIPT

the summary score. To make coefficients comparable across cities, we re-scaled the SVI so that it ranges from 0 to 1 in each city. We also used, for sensitivity analysis, a scaled version of the SVI that ranges from 0 to 1 across the whole sample. Last, we created quintiles both for the city-specific and the whole sample versions of the SVI. A higher value of the SVI signifies higher social vulnerability, either overall or by domain.

Analysis

We described basic neighborhood-level and city-level characteristics and graphically examined the relationship between the SVI and vaccination coverage using scatterplots with smoothed lowess lines and by computing vaccination coverage by quintile of the SVI. We then computed indicators of total relative and absolute inequities by estimating the ratio and difference between the top (defined as the neighborhood at the $90^{th}$ percentile of vaccination) vs bottom neighborhoods ($10^{th}$ percentile).

To describe relative and absolute SVI-related inequities we estimated the Relative Index of Inequality (RII) and the Slope Index of Inequality (SII). These indices represent the ratio or difference in vaccination coverage between the top (most vulnerable) and bottom (least vulnerable) parts of the SVI distribution, while accounting for the distribution across the full range of social vulnerability. We estimated the indices using a linear model at the neighborhood level, stratified by city, with log(vaccination coverage) (for the RII) or vaccination coverage (for the SII) as the outcomes, and the SVI as the main exposure. The SVI coefficient (exponentiated in the case of the RII) and associated 95% confidence intervals (CI) represent the RII or SII for the city. We adjusted these models by the % of the population of each neighborhood aged 18-44, 45-64, and 65 or above, but also show results unadjusted by age. We also run these models using the four SVI domains instead of the summary SVI. To address potential differences in the

ORIGINAL UNEDITED MANUSCRIPT

distribution of the SVI across cities we repeated the same models using the SVI scaled to the whole sample instead to each city separately.

To examine heterogeneity in the RII and SII, we used a multilevel linear model of neighborhoods nested in cities, with a random intercept for city and introduced the SVI as both a fixed and random coefficient. To test whether there was variability in these inequities we compared models with and without the SVI random slope using the likelihood ratio test. To assess whether there were geographical differences in these inequities, and how much of the variability in inequities was explained by geography, we added an interaction between the SVI and census region (using South as the reference, as it was the region with the largest number of observations).

All analyses were conducted using R v4.1 (R Foundation, Vienna, Austria). This analysis was approved by the Drexel University Institutional Review Board under number 2102008373.

**Results**

We included a total of 866 neighborhoods (ZCTAs) in 16 cities, representing 32.2 million residents (**Table 1**). The number of neighborhoods varied from a low of 11 in Long Beach (CA) to a high of 177 in New York City (NY); the median population by neighborhood was 33,486, ranging from 23,089 in Minneapolis (MN) to 46,612 in Chicago (IL). The 16 included cities were heterogeneous in size (from around 420,000 residents in Minneapolis to 8.42 million residents in New York City), racial-ethnic composition (from a low of 40% non-white residents in Minneapolis (MN) to a high of 76% in Houston (TX)), and socioeconomic status (poverty ranging from 8.7% in San Jose (CA) to 24.3% in Philadelphia (PA)). Through September 2021, a total of 18.8 million individuals (58.4% of the total population) had been fully vaccinated across

ORIGINAL UNEDITED MANUSCRIPT

the 16 cities. The online interactive dashboard (www.covid-inequities.info) contains maps for the SVI and vaccination coverage for the cities included in this study.

We found wide heterogeneity in the levels of vaccination between neighborhoods within cities (**Table 2**). Top/Bottom ratios varied from a low of 1.28 (neighborhoods at the 90th percentile of vaccination have 28% higher vaccination coverage than those at the 10th percentile of vaccination) in San Francisco and San Jose (CA) to a high of 1.71 (neighborhoods at the top having 71% higher vaccination coverage than those at the bottom) in Dallas (TX). Absolute differences between top and bottom neighborhoods also varied widely, from a low of 17-18% in San Jose, San Francisco, and Fort Worth (TX) to a high of 32% in New York City. We also found an overall monotonic association between SVI and vaccination coverage (**Figure 1 and Figure 2; and Web Figure 1 and Web Figure 2 for equivalent figures with the whole sample rescaled SVI**), so that in general areas with higher social vulnerability had lower vaccination coverage.

**Table 2** also shows the RII and SII for each city and overall, after adjusting for neighborhood-level age distribution. Overall, vaccination coverage in the most vulnerable areas was 0.75 times (RII=0.75, 95% CI 0.69 to 0.81) that of the least vulnerable areas, while the vaccination coverage was 16.2 percentage points lower in the most vs. least vulnerable areas (SII=-16.2%, 95% CI -20.3% to -12.1%). However, these inequities varied widely by city, as indicated by an improvement in model fit comparing multilevel models with and without random slopes (p-value for the log-likelihood ratio test=0.038 and 0.046 for the RII and SII, respectively, see **Web Table 2 and 3**). The correlation between random intercepts and random SVI slopes in multilevel models was very low for the RII ($\tau_{01} = -0.07$), indicating that relative inequities were similar across cities with different overall vaccination coverage, while we found a negative

ORIGINAL UNEDITED MANUSCRIPT

correlation for the SII ($\tau_{01} = -0.34$), indicating that cities with a higher vaccination coverage had wider absolute inequities.

We observed narrow, inexistent, or even inverted inequities in five cities, four of them in California (San Diego, Long Beach, San Francisco, and Oakland), and Philadelphia (PA). RIIs in these cities varied from 0.93 (95% CI 0.76 to 1.14) in San Diego to 1.13 (95% CI 0.97 to 1.32) in Oakland. Outside of these five cities, we observed wider relative inequities, with RIIs ranging from 0.61 (95% CI 0.43 to 0.85) in Fort Worth (TX) to 0.83 (95% CI 0.68 to 0.98) in San Jose (CA). These patterns generally held for absolute inequities, as measured by the SII (see **Web Figure 3** for a comparison between both measures, Spearman correlation coefficient between SII and RII=0.91). Specifically, we observed narrower or inverted absolute inequities in the same five cities, varying from -4.3% (95% CI -19.1% to 10.5%) in San Diego to 6.6% (95% CI -2.9% to 16.2%) in Oakland.

Results were relatively similar in models unadjusted for age (**Web Figure 4**; Spearman correlation coefficient between adjusted and unadjusted RIIs=0.65), with generally narrower inequities after age-adjustment. Models using the city-specific rescaled SVI vs the whole sample rescaled SVI showed virtually unchanged results (**Web Figure 5**; Spearman correlation coefficient between city-specific and nationwide SVI RIIs=0.99). We also tested whether there were regional differences in coverage, and found that, compared to the Southern US, cities in the West had a 27% narrower RII (exponentiated interaction coefficient=1.27, 95% CI 1.11 to 1.46), and an SII that was 12.7% percentage units closer to the null (interaction coefficient=12.7, 95% CI 5.6 to 19.8). Adding region and an interaction with the SVI explained 92% and 73% of the variability in inequities (**Web Table 2**).

ORIGINAL UNEDITED MANUSCRIPT

**Table 3** and **Table 4** show the RII and SII for the four SVI domains. In general, the four components of the SVI showed similar associations as the summary score, although the magnitudes differed by domain. Relative inequities were wider when using the socioeconomic status and household composition & disability domains (RII=0.74 and 0.75, respectively) as compared to the minority status & language and the housing type & transportation (RII=0.85 for both), with a similar pattern for absolute inequities (SII=-16.1%, -13.7%, -10.1%, and -11.1% for socioeconomic status, household composition & disability, minority status & language, and housing type & transportation). The heterogeneity in inequities across cities also varied by domain. Specifically, we found no improvement in model fit with random slopes (indicating no heterogeneity in inequities) for the minority status & language domain in relative inequities, and for the socioeconomic status domain for absolute inequities (see **Web Table 4**).

**Discussion**

We documented wide spatial inequities in COVID-19 vaccination through September 2021 in 16 large US cities. We found negative and heterogeneous associations between social vulnerability and vaccination coverage for all cities. Overall, coverage in areas of the highest social vulnerability was 0.75 times lower than the coverage in areas of the lowest social vulnerability, or 16% percentage points lower. However, these disparities were regionally heterogeneous, as cities in the West region tended to have narrower inequities compared to cities in other regions. We also observed that the social vulnerability domains of socioeconomic status and household composition & disability were more strongly associated with vaccination coverage, as compared to the domains of minority status & language and housing type & transportation.

Findings from this study mirror other reports examining inequities in COVID-19 positivity, incidence, and/or mortality by neighborhood(1, 4). This is highly problematic from an

ORIGINAL UNEDITED MANUSCRIPT

equity perspective, as areas that have been most affected have seen the lowest vaccination coverage, potentially leaving many residents in those areas vulnerable to COVID-19 infection and mortality, especially as immunity from infection may have a shorter duration than that of vaccines(18). In a modeling study exploring different strategies for vaccine prioritization, Wrigley-Field et al. found that prioritizing high-risk neighborhoods would have led to an overall lower mortality burden and narrower disparities than an age-based strategy alone (9). Our study shows that, regardless of the strategy used in each setting, inequities linked to social vulnerability persisted, highlighting the need for an implementation (or strengthening, if present) of neighborhood prioritization strategies.

While we cannot test what specific mechanisms are driving these inequities, we propose a few potential explanations that may have contributed to the large differences in vaccination coverage by social vulnerability across all cities. First, the eligibility schedule, which initially focused on healthcare workers and older adults. Both healthcare workers (especially physicians which have had the fastest vaccine uptake (19)) and older adults (9) are more likely to be white and of higher SES (20). Most eligibility schedules followed the Advisory Committee on Immunization Practices (ACIP) of the CDC (21), which was partially based on the National Academies of Sciences, Engineering and Medicine (NASEM) recommendations(5, 11). While the ACIP recommendations included ethical considerations (and among them, a mitigation of health inequities)(21), they did not recommend targeting specific areas with higher concentrations of low SES or minoritized populations, as the NASEM recommendations did(5). However, our results were adjusted by age (and this adjustment had relatively small effect on the estimates, see **Web Figure 4**), and, as of the date of this analysis, vaccines have been authorized for more than 9 months, and eligibility was opened to for five months (since April 2021),

ORIGINAL UNEDITED MANUSCRIPT

pointing to other reasons behind these inequities. In fact, the SVI domains with the strongest inequities (both relative and absolute) included the household composition & disability domain, which includes an indicator for the % of the population aged 65+.

Second, there are specific barriers to access for low SES population and minoritized populations. These include the location of vaccination sites(22), as spatial proximity to services can affect their use, as has been reported for COVID-19 testing(23). By March 2021, community districts in Brooklyn (New York City) with higher proportions of Black and Hispanic residents had fewer vaccinations sites compared to districts with higher proportion of white residents, while the number of residents per vaccination site was twice as much in high poverty areas compared to low poverty areas(22). For communities of color in particular, these spatial access issues are rooted in structural racism(24), especially considering hyper-segregation in some of the cities in this analysis(25). Moreover, though not required by the federal government, requests by some pharmacies and vaccine sites for identification, social security numbers, health insurance, and proof of employment(26), may have dissuaded undocumented migrants from getting vaccinated, while states have varied in their approach to providing information to the contrary(27). Other reports have also highlighted fear of side effects, including worry that side effects may lead to loss of work in people without paid sick leave(28), and difficulties taking time off to get the vaccine(28), especially given long or uncertain wait times that may force missed hours of work(29). Misinformation about eligibility and costs associated with the vaccine, especially misinformation targeted towards minoritized populations, may also contribute to disparities in vaccination coverage(30). For example, while Facebook enforced some rules around vaccine misinformation, these rules were not as quickly enforced for posts in Spanish (as compared to English)(31). Adoption of evidence-based strategies for communication

ORIGINAL UNEDITED MANUSCRIPT

may also help in combating this misinformation(32, 33). Last, a common narrative suggests that hesitancy is behind vaccination disparities. However, vaccine hesitancy has been repeatedly used as a scapegoat to justify lower vaccination coverage among communities of color(24). Studies about vaccine hesitancy often fail to contextualize the issue for communities of color, many of whom have longstanding and justified mistrust of medical research and institutions as well as concerns about how the vaccine works and how it might interact with treatments for pre-existing conditions(24), and fail to take into account that hesitancy varies by other key demographic factors within communities of color(19, 34). In fact, a recent study of vaccination uptake in 756 US counties highlighted the role of socioeconomic and political ideology in driving some of these disparities(35). This is consistent with our finding that there were stronger inequities (both absolute and relative) using the socioeconomic status domain as compared to the minority status & language domain. However, since our analysis is ecological in nature, we cannot rule out that disparities between population subgroups defined by race/ethnicity are driving our observed spatial inequities.

The second key finding of our study was the wide heterogeneity in spatial inequities across cities. Specifically, we found narrower disparities in cities in the West region of the US, all located in California (although it should be noted that Los Angeles had inequities similar to the overall pattern). California has implemented an extensive COVID-19 equity plan(36, 37), including a zip code prioritization plan that may explain these narrow disparities(38), although some criticisms have emerged on its implementation, including the use of zip codes instead of census tracts(39). A more focused study will be needed to ascertain the reasons behind the success of cities in California in avoiding wide disparities in COVID-19 vaccination. Outside of California, we observed wide heterogeneity in inequities, but without a clear geographical

ORIGINAL UNEDITED MANUSCRIPT

pattern. For example, in the case of Texas, Fort Worth, Dallas and Houston had some of the widest inequities, and Austin and San Antonio had narrower ones. Notably, while Dallas County (Texas) had planned to prioritize communities of color in their vaccine rollout, the state health department rejected this proposal(40).

Over time, cities varied and expanded the location of vaccination sites, including implementing pop-up sites in easily accessible locations, expanding mobile vaccination clinics, targeted vaccinations by zip code, allowing walk-ins and same day appointments, in-home vaccinations for the home-bound and their family and home health aides, and leveraging primary care clinics where people are already getting care(5, 41). Not all efforts were successful; for example the use of appointment links targeted for the most at-risk groups often led to misuse by other populations(42-44). While the increase in spatial availability of sites and the elimination of required appointments may help reduce inequities, other factors may persist as barriers to vaccination. Community engaged approaches developed by community-based organizations to reach populations with the most need and least access are critical, especially as the barriers to vaccination may differ from context to context. For example several cities have cooperated with community groups(45, 46), such as the Black Doctors COVID Consortium in Philadelphia(45), to address these inequities. Finding ways to scale up and expand these initiatives could be extremely valuable to reach large number of unvaccinated people in poor communities and communities of color. Other community organizations have helped with multi-lingual outreach, education, transportation, and vaccine sign ups(47), and will be critical in attempting to reach the unvaccinated population.

A limitation of our study is that we rely on aggregated surveillance data, which may not be complete. While we used data on neighborhood of residence, to our knowledge the data for

ORIGINAL UNEDITED MANUSCRIPT

Philadelphia, Chicago and New York City does not include residents who were vaccinated outside of their respective cities, while residents vaccinated outside their states of residence are not captured as vaccinated in the data we used. While we have no data on who was vaccinated outside their cities or states, considering the barriers outlined above, we speculate that individuals of high SES may be more likely to be vaccinated outside their cities, so this would bias our estimates of inequities towards the null. ZCTAs are very imperfect proxies for neighborhoods, but they represent a practical way to collect data during a public health emergency. Heterogeneity in the SVI (and its components) within zip codes may have led to underestimation of inequities. We also lacked longitudinal data for some of the cities included in our study, so we could not assess trends in these inequities; this type of data may be useful to evaluate some of the interventions outlined above. Last, given the cross-sectional, descriptive, and ecological nature of our study, caution should be used in drawing causal inferences or conclusions at the individual-level. For example, our adjustment for age was crude and indirect, adjusting for the age distribution of each neighborhood, as we had no data on vaccination by neighborhood and age. We also lacked data on coverage by neighborhood and race/ethnicity, so we could not explicitly examine disparities by population subgroup.

Conclusion

In summary, we found wide but heterogeneous spatial inequities in COVID-19 vaccination in 16 US cities, with areas of high social vulnerability having the lowest vaccination coverage. While we cannot infer a causal relationship between social vulnerability and vaccination coverage, the combination of these patterns with disproportionate impact of COVID-19 in these same neighborhoods(1) represents a worrying development as it may lead to even wider COVID-19 inequities in the future, regardless of their cause. The long history of income inequality and

ORIGINAL UNEDITED MANUSCRIPT

racial segregation in US cities, along with systematic disinvestment in poor and non-white neighborhoods(48), continues to affect health in these neighborhoods. Certainly, we need to learn from this pandemic experience in order to develop better strategies to improve efforts to deliver vaccines equitably in the future(41). Careful evaluation of the well-intentioned efforts many cities made to improve equity in vaccine access is needed. More generally however, the pandemic and our response to it has made it abundantly clear that addressing structural factors linked to income inequality, racism, and segregation will be fundamental to promoting population health and health equity across all health conditions. In October of 2020, well before any vaccines were approved for emergency use, a committee of the National Academies issued a consensus report with recommendations for vaccine distribution(11). A key focus of the report was the need to implement a national strategy that maximized equity and prioritized groups at highest risks for COVID-19, including minoritized populations, and low SES populations. The report specifically recommended prioritizing neighborhoods with low SVI within all vaccine distribution strategies. It is sobering, although not surprising, that despite prior knowledge and recommendations, vaccination coverage replicated underlying inequities once again.

ORIGINAL UNEDITED MANUSCRIPT

Table 1: Descriptive statistics [a] and vaccination outcomes in the 16 US cities included in this study through September 2021.

| City and State | Neighborhood Characteristics | | | City Characteristics | | | |
|---|---|---|---|---|---|---|---|
| | N | Population[b] | SVI, median (IQR) | Population[c] | % non-white | % Poverty | % Fully Vaccinated |
| Long Beach, CA | 11 | 39239 | 0.76 (0.46-0.92) | 0.47 | 71.8 | 16.8 | 57.3 |
| Los Angeles, CA | 79 | 37251 | 0.81 (0.52-0.90) | 3.97 | 71.5 | 18.0 | 59.2 |
| Oakland, CA | 16 | 30588 | 0.69 (0.54-0.89) | 0.43 | 71.7 | 16.7 | 67.2 |
| San Diego, CA | 38 | 42585 | 0.50 (0.34-0.79) | 1.41 | 57.2 | 12.8 | 65.0 |
| San Francisco, CA | 26 | 31449 | 0.57 (0.47-0.73) | 0.87 | 59.5 | 10.3 | 74.0 |
| San Jose, CA | 31 | 36975 | 0.54 (0.40-0.66) | 1.03 | 74.3 | 8.7 | 71.9 |
| Chicago, IL | 58 | 46612 | 0.71 (0.46-0.93) | 2.71 | 66.7 | 18.4 | 55.9 |
| Indianapolis, IN | 35 | 28705 | 0.75 (0.37-0.91) | 0.86 | 45.5 | 18.0 | 50.2 |
| Minneapolis, MN | 22 | 23089 | 0.62 (0.49-0.84) | 0.42 | 40.0 | 19.1 | 63.2 |
| New York City, NY | 177 | 42726 | 0.75 (0.60-0.89) | 8.42 | 67.9 | 17.9 | 62.8 |
| Philadelphia, PA | 46 | 34022 | 0.86 (0.65-0.96) | 1.58 | 65.5 | 24.3 | 44.5 |
| Austin, TX | 45 | 24313 | 0.48 (0.27-0.75) | 0.95 | 51.7 | 13.2 | 62.2 |
| Dallas, TX | 57 | 26867 | 0.77 (0.39-0.93) | 1.33 | 71.0 | 18.9 | 50.2 |
| Fort Worth, TX | 43 | 26563 | 0.69 (0.42-0.88) | 0.87 | 60.8 | 14.5 | 48.0 |

ORIGINAL UNEDITED MANUSCRIPT

| | | | | | | | |
|---|---|---|---|---|---|---|---|
| Houston, TX | 122 | 34032 | 0.76 (0.52-0.92) | 2.31 | 75.6 | 20.1 | 53.9 |
| San Antonio, TX | 60 | 29923 | 0.83 (0.45-0.98) | 1.51 | 75.3 | 17.8 | 55.0 |
| Total[d] | 866 | 33486 | 0.74 (0.47-0.91) | 32.1 | 67.1 | 17.5 | 58.4 |

SVI=social vulnerability index.
[a] Population, poverty, and race/ethnicity descriptive statistics correspond to the 2015-2019 5-year ACS.
**[b]** Nationwide social vulnerability index, median [Q1-Q3].
**[c]** Total city population in millions.
[d] Total refers to the whole sample.

ORIGINAL UNEDITED MANUSCRIPT

**Table 2: Total and age-adjusted social vulnerability index (SVI)-related inequities (and 95% CI) in full COVID-19 vaccination by neighborhood in 16 US cities through September 2021.**

| City and State | Total Inequities[a] | | | | SVI-Related Inequities[b] | | | |
|---|---|---|---|---|---|---|---|---|
| | P90 | P10 | Ratio | Difference | RII | 95% CI | SII | 95% CI |
| Long Beach, CA | 65.3 | 46.6 | 1.40 | 18.6 | 0.99 | 0.86,1.15 | -0.72 | -8.08,6.64 |
| Los Angeles, CA | 71.8 | 46.5 | 1.54 | 25.3 | 0.75 | 0.65,0.87 | -18.19 | -27.93,-8.45 |
| Oakland, CA | 79.4 | 56.7 | 1.40 | 22.7 | 1.13 | 0.97,1.32 | 6.61 | -2.93,16.15 |
| San Diego, CA | 79.0 | 53.3 | 1.48 | 25.7 | 0.93 | 0.76,1.14 | -4.31 | -19.12,10.50 |
| San Francisco, CA | 82.8 | 64.9 | 1.28 | 17.9 | 1.00 | 0.78,1.28 | 1.66 | -16.41,19.73 |
| San Jose, CA | 78.7 | 61.4 | 1.28 | 17.3 | 0.83 | 0.73,0.94 | -14.32 | -23.94,-4.71 |
| Chicago, IL | 67.3 | 40.9 | 1.64 | 26.3 | 0.73 | 0.44,1.21 | -13.75 | -28.04,0.53 |
| Indianapolis, IN | 62.7 | 38.1 | 1.65 | 24.6 | 0.65 | 0.58,0.74 | -22.19 | -28.45,-15.93 |
| Minneapolis, MN | 72.6 | 49.9 | 1.46 | 22.8 | 0.72 | 0.59,0.88 | -18.85 | -29.42,-8.27 |
| New York City, NY | 80.1 | 48.6 | 1.65 | 31.6 | 0.82 | 0.73,0.92 | -13.13 | -21.04,-5.22 |
| Philadelphia, PA | 57.6 | 35.3 | 1.63 | 22.3 | 0.98 | 0.79,1.21 | -0.51 | -10.68,9.65 |
| Austin, TX | 72.4 | 46.2 | 1.57 | 26.3 | 0.79 | 0.65,0.96 | -16.22 | -26.29,-6.15 |
| Dallas, TX | 65.5 | 38.2 | 1.71 | 27.3 | 0.68 | 0.59,0.79 | -16.90 | -25.12,-8.68 |
| Fort Worth, TX | 58.7 | 40.8 | 1.44 | 17.8 | 0.61 | 0.43,0.86 | -21.37 | -32.36,-10.38 |
| Houston, TX | 70.7 | 43.2 | 1.64 | 27.5 | 0.65 | 0.59,0.72 | -25.65 | -32.12,-19.18 |
| San Antonio, TX | 67.2 | 44.5 | 1.51 | 22.6 | 0.82 | 0.68,0.99 | -17.15 | -24.71,-9.59 |
| Total[c] | 75.0 | 43.3 | 1.73 | 31.7 | 0.75 | 0.69,0.81 | -16.20 | -20.27,-12.13 |

P90=90$^{th}$ percentile, P10=10$^{th}$ percentile, SVI=social vulnerability index, RII=Relative Index of Inequality, SII=Slope Index of Inequality.

[a] total inequities represent the difference or ratio between neighborhoods (ZCTA) at the 90$^{th}$ vs 10$^{th}$ percentile.

[b] SVI-related inequities are the relative index of inequality and the slope index of inequality, both adjusted for the % of the population of the neighborhood aged 18 to 44, 45 to 64, and 65 or above, and are the best linear unbiased predictor (BLUP) of a multilevel model with a random intercept for city and random slope for the SVI.

[c] Total represents the entire sample of neighborhoods across all cities; for the RII and SII it represents the fixed effect in the same multilevel model.

ORIGINAL UNEDITED MANUSCRIPT

**Table 3: Age-adjusted social vulnerability index (SVI) domain-related relative index of inequality (and 95% CI) in full COVID-19 vaccination by neighborhood in 16 US cities through September 2021.**

| City and State | Socioeconomic Status | | Household Composition and Disability | | Minority Status and Language | | Housing Type and Transportation | |
|---|---|---|---|---|---|---|---|---|
| | RII [a] | 95% CI | RII [a] | 95% CI | RII [a] | 95% CI | RII [a] | 95% CI |
| Long Beach, CA | 1.00 | 0.88,1.14 | 0.97 | 0.86,1.09 | 1.05 | 0.92,1.20 | 1.06 | 0.93,1.20 |
| Los Angeles, CA | 0.77 | 0.68,0.88 | 0.78 | 0.67,0.91 | 0.76 | 0.64,0.91 | 0.73 | 0.61,0.88 |
| Oakland, CA | 1.14 | 0.94,1.39 | 1.17 | 0.99,1.37 | 1.11 | 0.95,1.29 | 1.43 | 1.17,1.73 |
| San Diego, CA | 0.98 | 0.80,1.20 | 0.97 | 0.78,1.21 | 1.01 | 0.78,1.31 | 0.87 | 0.69,1.10 |
| San Francisco, CA | 0.92 | 0.73,1.15 | 1.02 | 0.78,1.34 | 1.19 | 0.93,1.51 | 1.04 | 0.77,1.41 |
| San Jose, CA | 0.89 | 0.79,0.99 | 1.12 | 0.94,1.32 | 0.76 | 0.65,0.89 | 0.81 | 0.72,0.92 |
| Chicago, IL | 0.81 | 0.51,1.28 | 0.67 | 0.43,1.04 | 1.19 | 0.73,1.93 | 0.79 | 0.50,1.24 |
| Indianapolis, IN | 0.65 | 0.59,0.72 | 0.57 | 0.51,0.64 | 0.62 | 0.40,0.94 | 0.67 | 0.55,0.82 |
| Minneapolis, MN | 0.67 | 0.55,0.81 | 0.71 | 0.62,0.80 | 0.82 | 0.68,0.98 | 0.85 | 0.65,1.10 |
| New York City, NY | 0.87 | 0.80,0.96 | 0.78 | 0.71,0.86 | 0.93 | 0.73,1.18 | 0.99 | 0.85,1.15 |
| Philadelphia, PA | 0.89 | 0.73,1.09 | 0.82 | 0.67,1.01 | 1.22 | 1.01,1.46 | 1.00 | 0.86,1.16 |
| Austin, TX | 0.76 | 0.63,0.92 | 0.63 | 0.49,0.81 | 0.92 | 0.76,1.12 | 0.90 | 0.72,1.12 |
| Dallas, TX | 0.70 | 0.62,0.80 | 0.63 | 0.54,0.74 | 0.63 | 0.50,0.78 | 0.76 | 0.62,0.93 |
| Fort Worth, TX | 0.59 | 0.45,0.79 | 0.53 | 0.36,0.76 | 0.69 | 0.45,1.05 | 0.71 | 0.48,1.06 |
| Houston, TX | 0.68 | 0.62,0.74 | 0.61 | 0.54,0.69 | 0.67 | 0.54,0.83 | 0.72 | 0.64,0.82 |
| San Antonio, TX | 0.81 | 0.67,0.97 | 0.79 | 0.64,0.99 | 0.81 | 0.63,1.04 | 0.81 | 0.66,1.00 |
| Total[b] | 0.74 | 0.69,0.80 | 0.75 | 0.67,0.84 | 0.85 | 0.77,0.94 | 0.85 | 0.77,0.93 |

RII: relative index of inequality,

[a] Adjusted for the % of the population of the neighborhood (ZCTA) aged 18 to 44, 45 to 64, and 65 or above.

[b] Total refers to the overall effect across all cities (fixed effect of the multilevel model).

ORIGINAL UNEDITED MANUSCRIPT

**Table 4: Age-adjusted social vulnerability index (SVI) domain-related slope index of inequality (and 95% CI) in full COVID-19 vaccination by neighborhood in 16 US cities through September 2021.**

| City and State | Socioeconomic Status | | Household Composition and Disability | | Minority Status and Language | | Housing Type and Transportation | |
|---|---|---|---|---|---|---|---|---|
| | SII [a] | 95% CI | SII [a] | 95% CI | SII [a] | 95% CI | SII [a] | 95% CI |
| Long Beach, CA | -0.54 | -6.93,5.85 | -1.93 | -7.93,4.08 | 2.15 | -4.48,8.78 | 2.41 | -3.88,8.69 |
| Los Angeles, CA | -16.69 | -25.62,-7.77 | -13.21 | -23.80,-2.62 | -18.32 | -30.41,-6.23 | -21.17 | -33.84,-8.49 |
| Oakland, CA | 7.47 | -4.65,19.59 | 8.09 | -2.04,18.23 | 4.99 | -4.33,14.31 | 20.19 | 7.79,32.59 |
| San Diego, CA | -0.74 | -15.62,14.13 | -1.17 | -17.35,15.00 | 0.80 | -18.12,19.72 | -8.92 | -25.97,8.14 |
| San Francisco, CA | -4.41 | -21.11,12.29 | 4.20 | -15.57,23.98 | 13.34 | -4.25,30.92 | 2.46 | -19.75,24.68 |
| San Jose, CA | -8.74 | -17.06,-0.43 | 9.68 | -2.72,22.07 | -21.05 | -32.70,-9.40 | -15.89 | -24.87,-6.92 |
| Chicago, IL | -9.27 | -22.65,4.11 | -14.27 | -26.83,-1.70 | 0.05 | -14.18,14.29 | -9.98 | -23.19,3.23 |
| Indianapolis, IN | -21.77 | -26.75,-16.79 | -28.37 | -34.58,-22.16 | -26.22 | -47.66,-4.77 | -20.80 | -30.66,-10.95 |
| Minneapolis, MN | -23.13 | -33.17,-13.10 | -18.08 | -25.51,-10.64 | -10.62 | -20.55,-0.69 | -9.50 | -23.90,4.91 |
| New York City, NY | -8.96 | -15.22,-2.69 | -15.67 | -22.17,-9.17 | -6.26 | -22.41,9.89 | -0.25 | -10.32,9.81 |
| Philadelphia, PA | -5.46 | -14.75,3.83 | -8.53 | -18.59,1.53 | 10.23 | 1.62,18.83 | 0.78 | -6.13,7.70 |
| Austin, TX | -18.20 | -27.79,-8.60 | -23.34 | -37.42,-9.26 | -9.51 | -19.83,0.81 | -10.29 | -22.04,1.46 |
| Dallas, TX | -15.45 | -22.64,-8.26 | -18.89 | -28.16,-9.62 | -26.16 | -37.41,-14.90 | -11.48 | -21.95,-1.01 |
| Fort Worth, TX | -19.59 | -28.96,-10.21 | -25.40 | -37.43,-13.38 | -15.79 | -30.08,-1.50 | -17.70 | -30.85,-4.55 |
| Houston, TX | -23.10 | -29.02,-17.18 | -28.46 | -36.42,-20.51 | -27.39 | -40.60,-14.17 | -18.32 | -26.30,-10.34 |
| San Antonio, TX | -18.53 | -26.29,-10.76 | -18.80 | -28.14,-9.46 | -21.13 | -31.55,-10.70 | -17.44 | -26.19,-8.68 |
| Total[b] | -16.05 | -19.51,-12.60 | -13.72 | -19.55,-7.89 | -10.15 | -16.45,-3.84 | -11.14 | -16.63,-5.65 |

SII: slope index of inequality.

[a] Adjusted for the % of the population of the neighborhood (ZCTA) aged 18 to 44, 45 to 64, and 65 or above.

[b] Total refers to the overall effect across all cities (fixed effect of the multilevel model).

ORIGINAL UNEDITED MANUSCRIPT

**Figures Titles/Legends**

**Figure 1:** Scatterplots showing the relationship between the social vulnerability index and COVID-19 vaccination coverage in neighborhoods of 16 US cities through September 2021. Solid lines are lowess smoothers for each city separately. The SVI has been rescaled for each city to fit in a 0-1 range. Neighborhoods are proxied by zip code tabulation areas (ZCTA). A) Long Beach, CA; B) Los Angeles, CA; C) Oakland, CA; D) San Diego, CA; E) San Francisco, CA; F) San Jose, CA; G) Chicago, IL; H) Indianapolis, IN; I) Minneapolis, MN; J) New York City, NY; K) Philadelphia, PA; L) Austin, TX; M) Dallas, TX; N) Fort Worth, TX; O) Houston, TX; P) San Antonio, TX.

**Figure 2:** COVID-19 Vaccination coverage by social vulnerability quintile (city-specific) and Census Region in 16 large US cities through September 2021. A) Midwest; B) Northeast; C) West; D) South.

ORIGINAL UNEDITED MANUSCRIPT

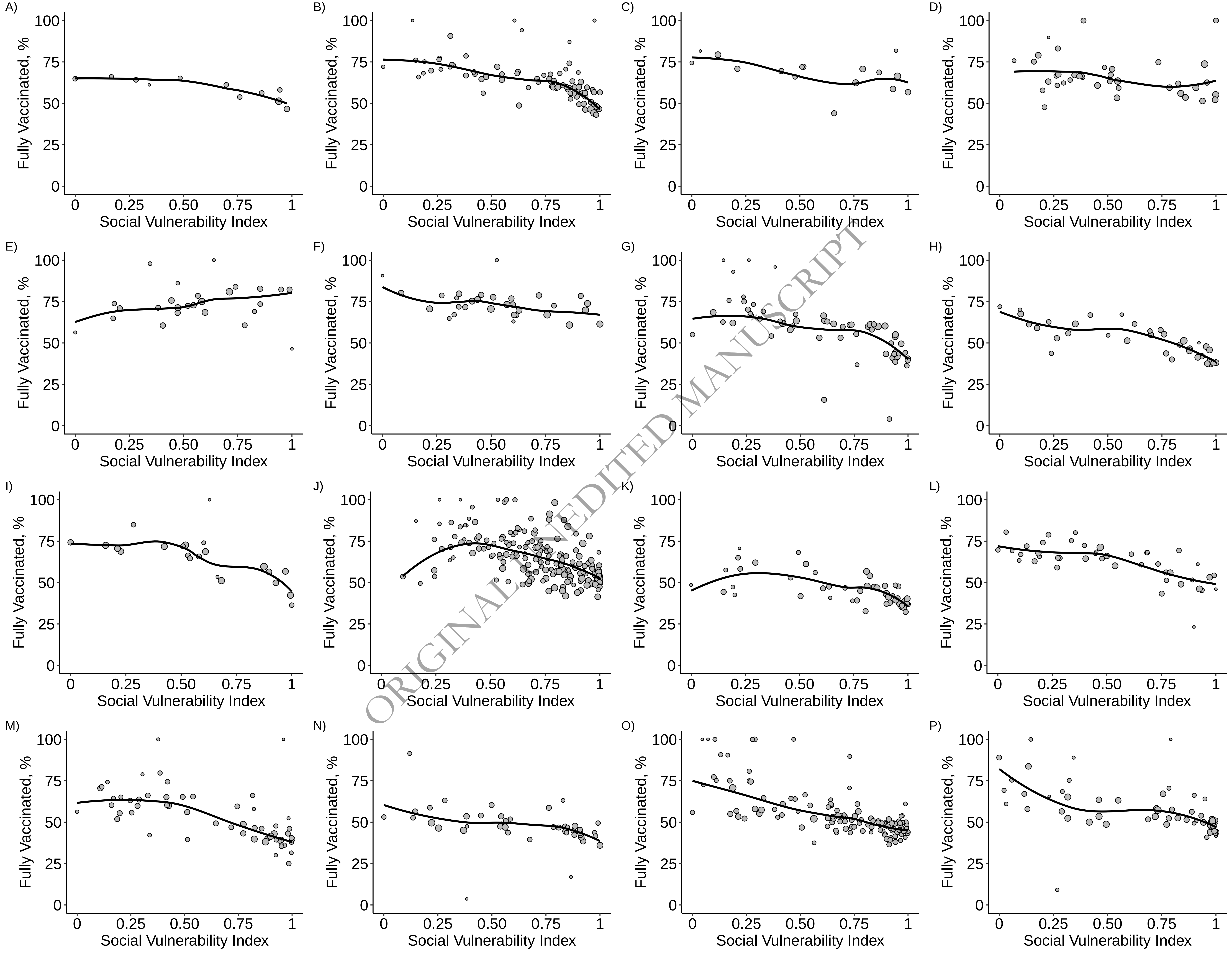

A)
B)
C)
D)
E)
F)
G)
H)
I)
J)
K)
L)
M)
N)
O)
P)
Fully Vaccinated, %
Social Vulnerability Index
ORIGINAL UNEDITED MANUSCRIPT

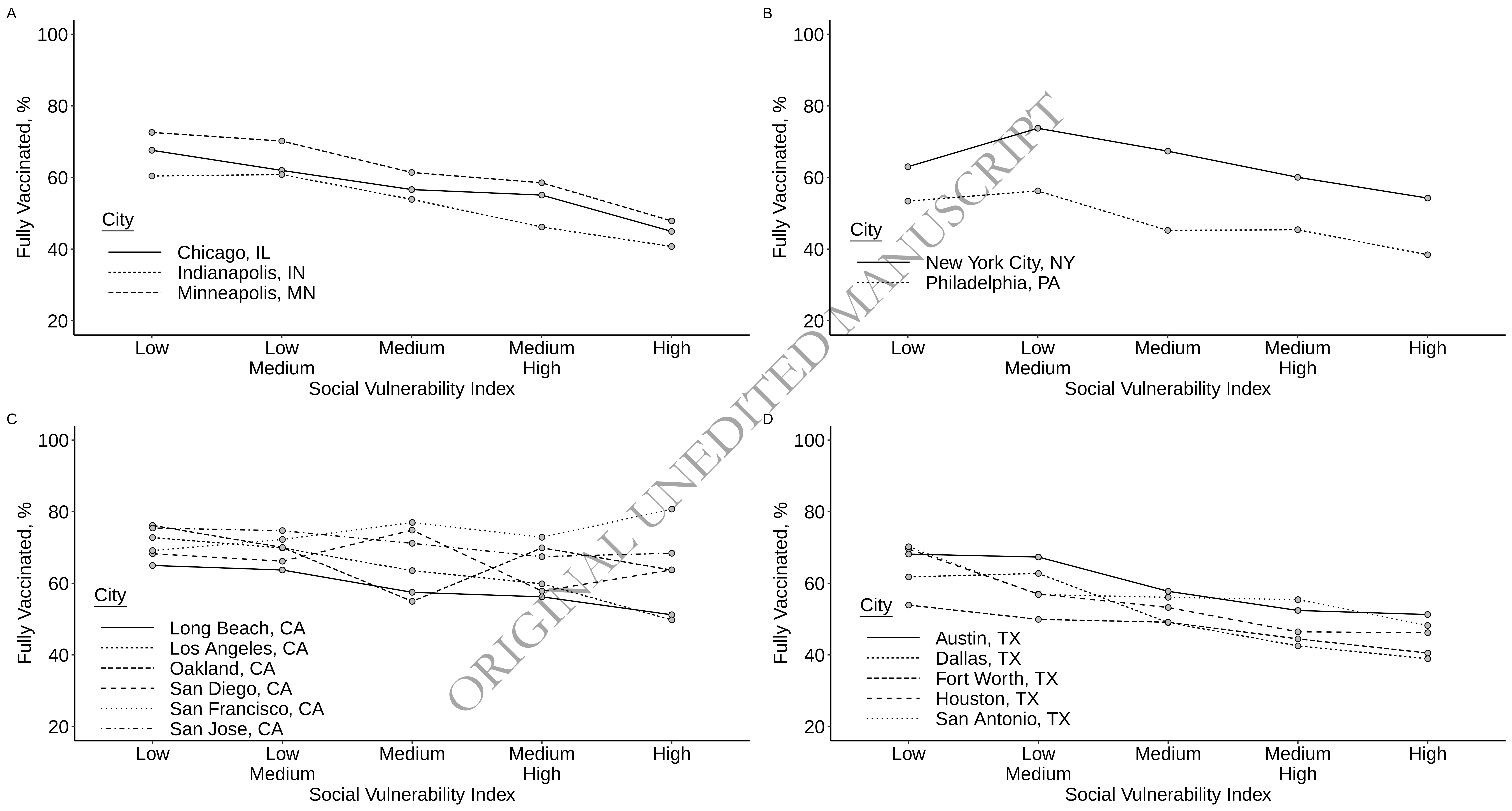

ORIGINAL UNEDITED MANUSCRIPT

## Supplementary Material

Web_Material_kwac076

