# Supplementary material for: Heterogeneity in Spatial Inequities in COVID-19 Vaccination Across 16 Large US Cities
Source: Am J Epidemiol. 2022 Apr 22;191(9):1546–56. doi: 10.1093/aje/kwac076 (PMC9047229; doi:10.1093/aje/kwac076)
Supplement: Web_Material_kwac076 [file web_material_kwac076.zip › kwac076 Bilal Web Material Final.pdf]

WEB MATERIAL

Heterogeneity in Spatial Inequities in COVID-19 Vaccination Across 16 Large US Cities

Usama Bilal, Pricila H. Mullachery, Alina Schnake-Mahl, Heather Rollins,Edwin McCulley, Jennifer Kolker, Sharrelle Barber, Ana V. Diez Roux

Table of Contents

Web Table 1: Data sources for this study by city.

Web Table 2: Fixed and random components of multilevel models for the relative and slope indices of inequality

Web Table 3: Model fit statistics and comparison of models with and without random slopes for the SVI

Web Table 4: Fixed and random components of multilevel models for the four social vulnerability index domains

Web Figure 1: Scatterplots showing the relationship between the social vulnerability index and COVID-19 vaccination coverage in ZCTAs of 16 US cities through September 2021.

Web Figure 2: COVID-19 Vaccination coverage by social vulnerability quintile (whole sample) in 16 large US cities through September 2021.

Web Figure 3: Comparison of relative and slope index of inequalities in COVID-19 vaccination coverage by social vulnerability index in 16 large US cities through September 2021

Web Figure 4: Comparison of age-adjusted and unadjusted relative index of inequalities in COVID-19 vaccination coverage by social vulnerability index in 16 large US cities through September 2021

Web Figure 5: Comparison of relative indices of inequalities in COVID-19 vaccination coverage by social vulnerability index (city-standardized vs whole sample) in 16 large US cities through September 2021

Web Table 1: Data sources for this study by city.

City	Source	URL
Long Beach, CA<br>Los Angeles, CA<br>Oakland, CA<br>San Diego, CA<br>San Francisco, CA<br>San Jose, CA	California Department of Public Health	https://github.com/datadesk/california-coronavirus-data
Chicago, IL	City of Chicago Department of Public Health	https://data.cityofchicago.org/
Indianapolis, IN	Indiana State Department of Health	https://hub.mph.in.gov/organization/indiana-state-department-of-health
New York City, NY	New York Department of Health and Mental Hygiene	https://github.com/nychealth/covid-vaccine-data
Minneapolis, MN	Minnesota Department of Health	https://www.mn.gov/covid19/vaccine/data/index.jsp
Philadelphia, PA	Philadelphia Department of Public Health	https://www.opendataphilly.org/
Austin, TX<br>Dallas, TX<br>Fort Worth, TX<br>Houston, TX<br>San Antonio, TX	Texas department of State Health Services	https://www.dshs.texas.gov/coronavirus/additionaldata/

Footnote: all compiled data is available at www.covid-inequities.info

Web Table 2: Fixed and random components of multilevel models for the relative and slope indices of inequality

		Relative Index of Inequality*			Slope Index of Inequality		
		Model without random slope	Main model	Main model + interaction with region	Model without random slope	Main model	Main model + interaction with region
Fixed	SVI (95% CI)	0.73 (0.69;0.77)	0.75 (0.69;0.81)	0.70 (0.64;0.76)	-17.8 (-20.5;-15.1)	-16.2 (-20.3;-12.1)	-21.1 (-25.7;-16.5)
	Age18_44	2.82 (2.23;3.58)	2.65 (2.09;3.36)	2.68 (2.12;3.40)	74.7 (63.5;85.8)	72.2 (61;83.5)	73.7 (62.5;84.8)
	Age45_64	4.95 (2.99;8.18)	4.61 (2.79;7.62)	4.44 (2.69;7.33)	115.4 (91.7;139.2)	110.6 (86.9;134.4)	109.7 (86.1;133.3)
	Age65+	4.04 (2.72;5.99)	4.01 (2.7;5.94)	4.26 (2.86;6.34)	77.4 (58.7;96.1)	77.8 (59;96.6)	82.2 (63.5;100.9)
	South x SVI			1 (Ref.)			1 (Ref.)
	Midwest x SVI			0.88 (0.75;1.03)			-2.5 (-10.8;5.8)
	West x SVI			1.27 (1.11;1.46)			12.7 (5.6;19.8)
	Northeast x SVI			1.07 (0.92;1.25)			6 (-2.4;14.4)
Random	τ_{00}	0.0133	0.0097	0.013	37.975	43.834	58.449
	τ_{11}		0.014	0.0011		35.425	9.511
	τ_{01}		-0.0723	-0.5624		-0.339	-1
	σ^2	0.046	0.0451	0.0449	102.506	100.052	99.653

Footnote: τ_{00} corresponds to the variance of the random intercept, τ_{11} corresponds to the variance of the random SVI slope, $\tau_{01}$is their correlation, and σ^2 is the variance of the level 1 residuals; *RII coefficients are exponentiated

Web Table 3: Model fit statistics and comparison of models with and without random slopes for the SVI

Model	Without Random Slope	With Random Slope	p-val of LRT*
**RII: SVI**	logLik=77.8; df=7	logLik=81.5; df=9	0.038
**RII: SVI1**	logLik=89.4; df=7	logLik=93.8; df=9	0.016
**RII: SVI2**	logLik=80.5; df=7	logLik=94.5; df=9	<0.001
**RII: SVI3**	logLik=33.5; df=7	logLik=35.7; df=9	0.155
**RII: SVI4**	logLik=35.6; df=7	logLik=39.3; df=9	0.036
**SII: SVI**	logLik=-3242.5; df=7	logLik=-3238.9; df=9	0.046
**SII: SVI1**	logLik=736.6; df=7	logLik=739.4; df=9	0.097
**SII: SVI2**	logLik=712.9; df=7	logLik=729.3; df=9	<0.001
**SII: SVI3**	logLik=666.4; df=7	logLik=674.4; df=9	<0.001
**SII: SVI4**	logLik=671.5; df=7	logLik=680; df=9	<0.001

Footnote: *LRT=log likelihood test testing the null hypothesis that the model with random slope has no improvement in model fit compared to the model without random slope. SVI1=socioeconomic status, SVI2=household composition and disability; SVI3=minority status and language; SVI4=housing type and transportation. df=degrees of freedom; logLik=log-likelihood

Web Table 4: Fixed and random components of multilevel models for the four social vulnerability index domains

		RII model*				SII model			
		**SVI 1**	**SVI 2**	**SVI 3**	**SVI 4**	**SVI 1**	**SVI 2**	**SVI 3**	**SVI 4**
**Fixed (95% CI)**	**SVI**	0.74 (0.69;0.8)	0.75 (0.67;0.84)	0.85 (0.77;0.94)	0.85 (0.77;0.93)	-16.05 (-19.51;-12.6)	-13.72 (-19.55;-7.89)	-10.15 (-16.45;-3.84)	-11.14 (-16.63;-5.65)
	**Age18_44**	2.41 (1.9;3.06)	1.99 (1.54;2.56)	3.66 (2.85;4.69)	4.88 (3.88;6.13)	66.92 (55.6;78.25)	60.55 (48.37;72.74)	86.96 (75.09;98.83)	106.11 (95.22;117)
	**Age45_64**	4.3 (2.62;7.08)	4.33 (2.62;7.15)	9.16 (5.48;15.31)	9.56 (5.76;15.87)	106.86 (83.29;130.43)	113.47 (89.47;137.47)	143.82 (119.25;168.39)	150.05 (125.96;174.15)
	**Age65+**	2.86 (1.91;4.27)	5.02 (3.44;7.33)	4.83 (3.15;7.41)	6.83 (4.56;10.24)	59.69 (40.34;79.04)	92.47 (74.34;110.59)	84.7 (64.19;105.21)	107.2 (87.94;126.46)
**Random**	$\boldsymbol{\tau_{00}}$	0.006	0.008	0.016	0.031	0.003	0.002	0.010	0.010
	$\boldsymbol{\tau_{11}}$	0.009	0.038	0.017	0.018	0.002	0.010	0.010	0.007
	$\boldsymbol{\tau_{01}}$	0.471	-0.087	-0.423	-0.756	-0.117	-0.044	-0.737	-0.790
	$\boldsymbol{\sigma^2}$	0.044	0.043	0.050	0.050	0.010	0.010	0.011	0.011

Footnote: τ_{00} corresponds to the variance of the random intercept, τ_{11} corresponds to the variance of the random SVI slope, $\tau_{01}$is their correlation, and σ^2 is the variance of the level 1 residuals. *: RII fixed coefficients are exponentiated; all models are adjusted for the % of the population of the ZCTA aged 18 to 44, 45 to 64, and 65 or above; SVI1=socioeconomic status, SVI2=household composition and disability; SVI3=minority status and language; SVI4=housing type and transportation

Web Figure 1: Scatterplots showing the relationship between the social vulnerability index and COVID-19 vaccination coverage in ZCTAs of 16 US cities through September 2021.

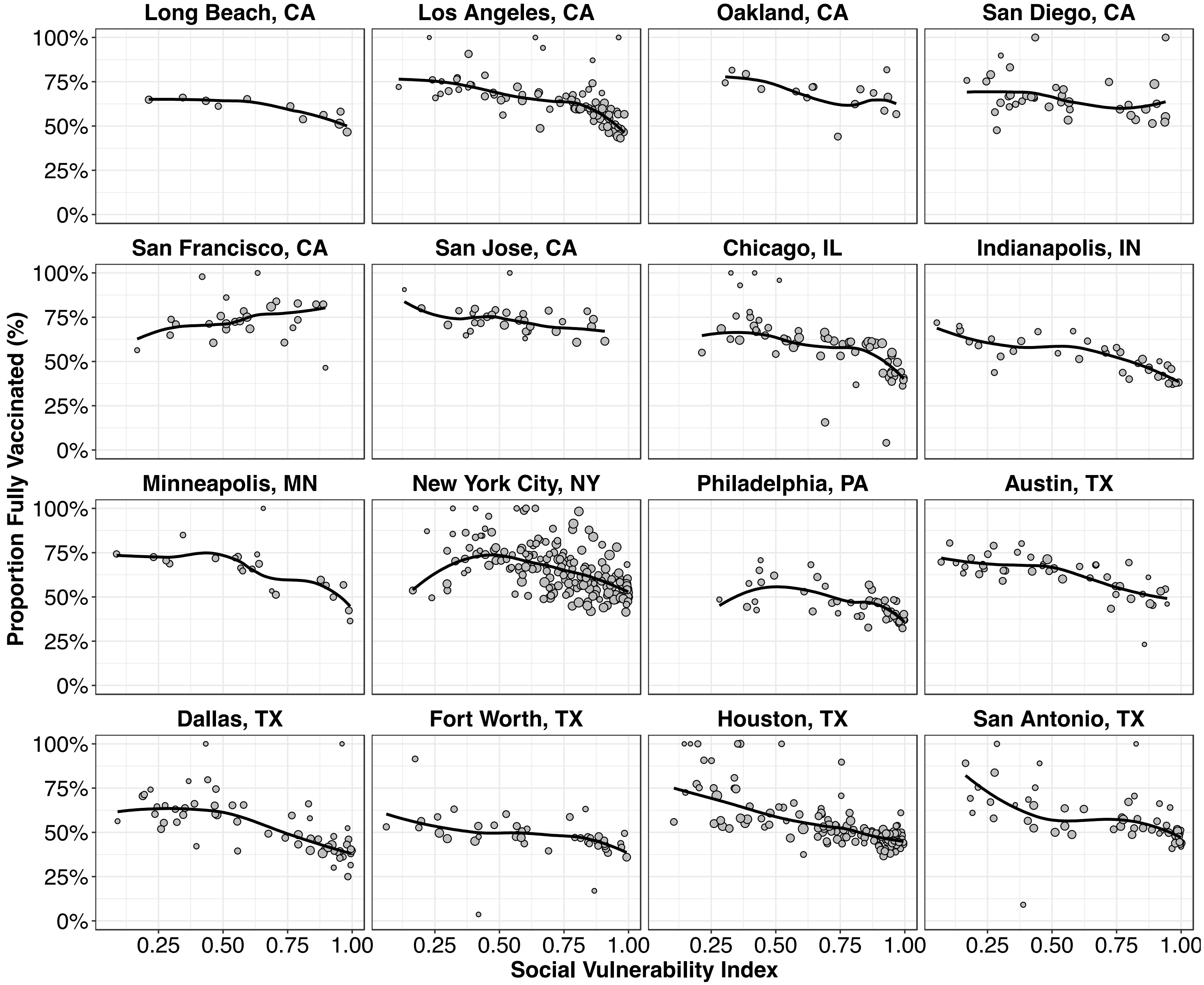

Footnote: solid lines are lowess smoothers for each city separately. The SVI has been re-scaled for the whole sample.

Web Figure 2: COVID-19 Vaccination coverage by social vulnerability quintile (whole sample) in 16 large US cities through September 2021.

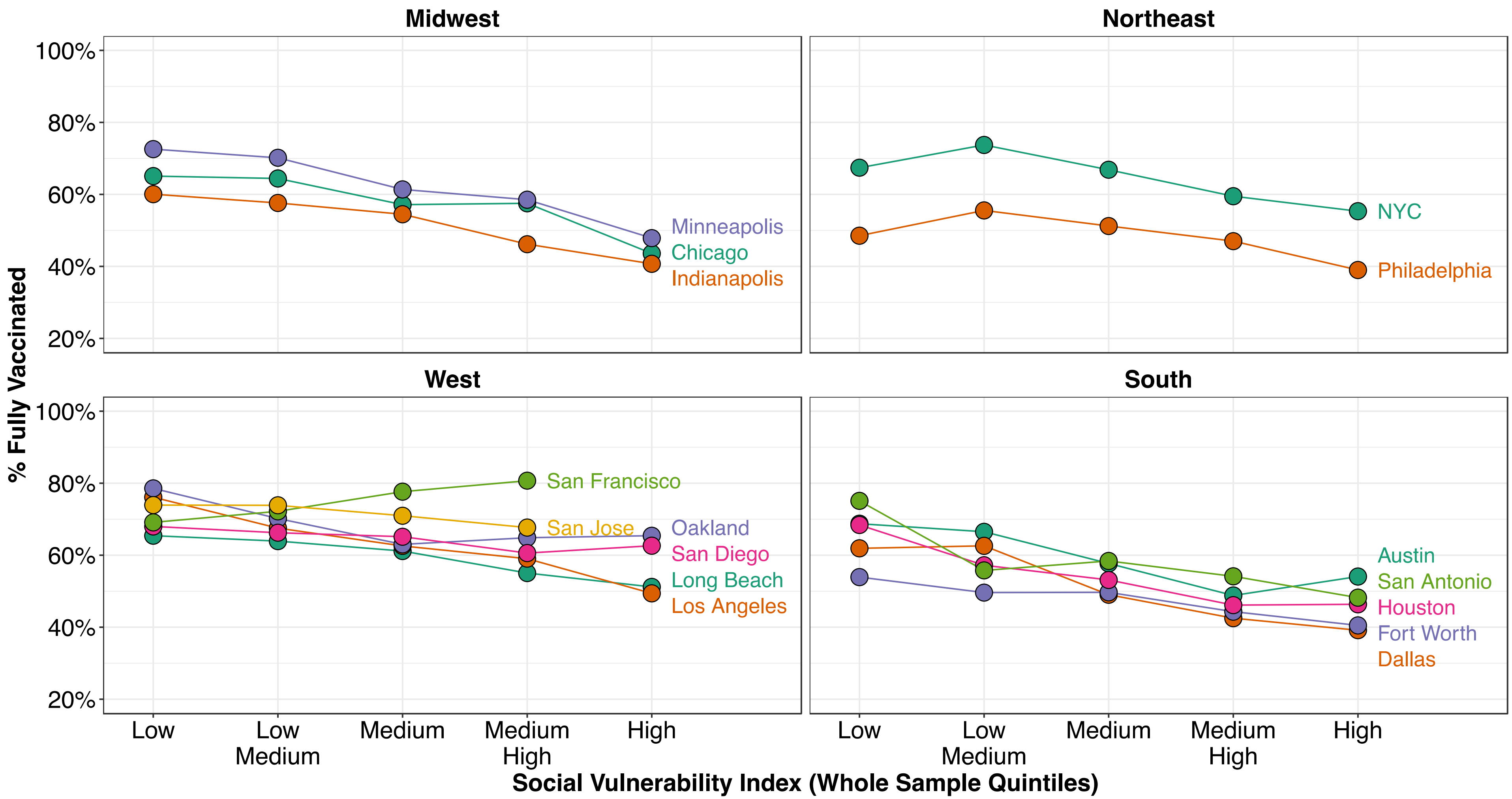

Footnote: social vulnerability index quintiles correspond to the whole sample.

Web Figure 3: Comparison of relative and slope index of inequalities in COVID-19 vaccination coverage by social vulnerability index in 16 large US cities through September 2021

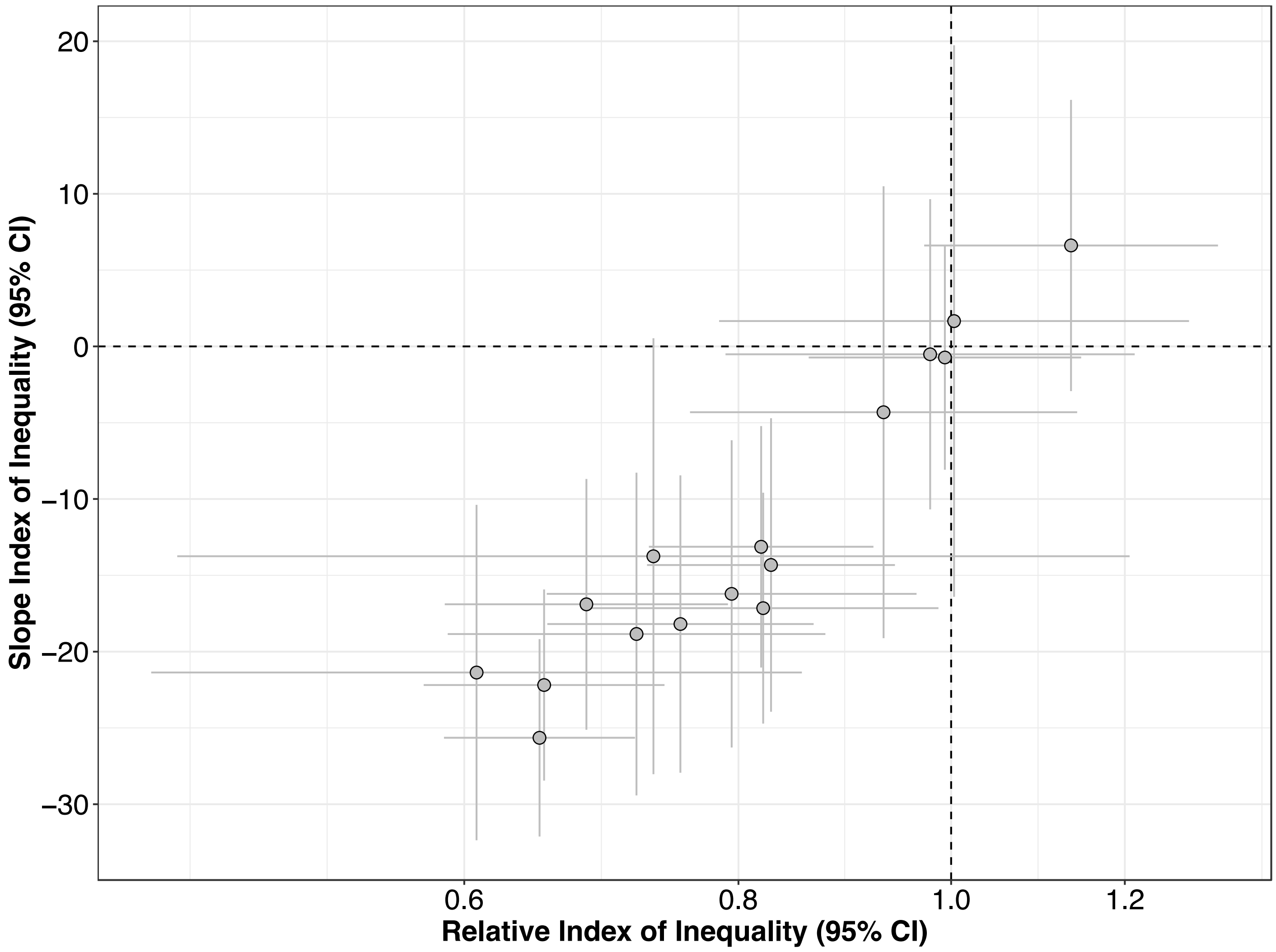

Footnote: both models adjusted for the % of the population of the ZCTA aged 18 to 44, 45 to 64, and 65 or above.

Web Figure 4: Comparison of age-adjusted and unadjusted relative index of inequalities in COVID-19 vaccination coverage by social vulnerability index in 16 large US cities through September 2021

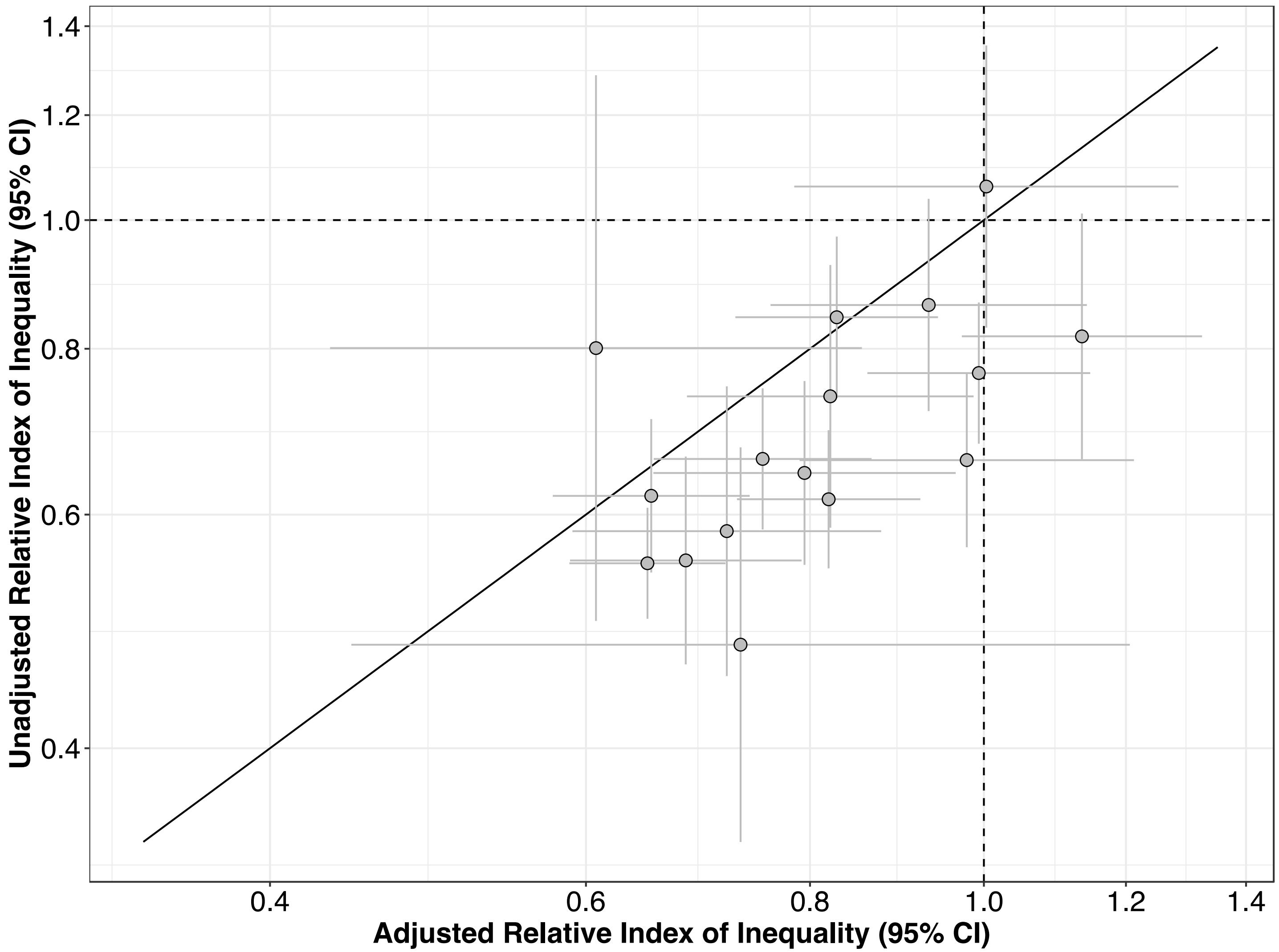

Footnote: adjusted model controls for the % of the population of the ZCTA aged 18 to 44, 45 to 64, and 65 or above.

Web Figure 5: Comparison of relative indices of inequalities in COVID-19 vaccination coverage by social vulnerability index (city-standardized vs whole sample) in 16 large US cities through September 2021

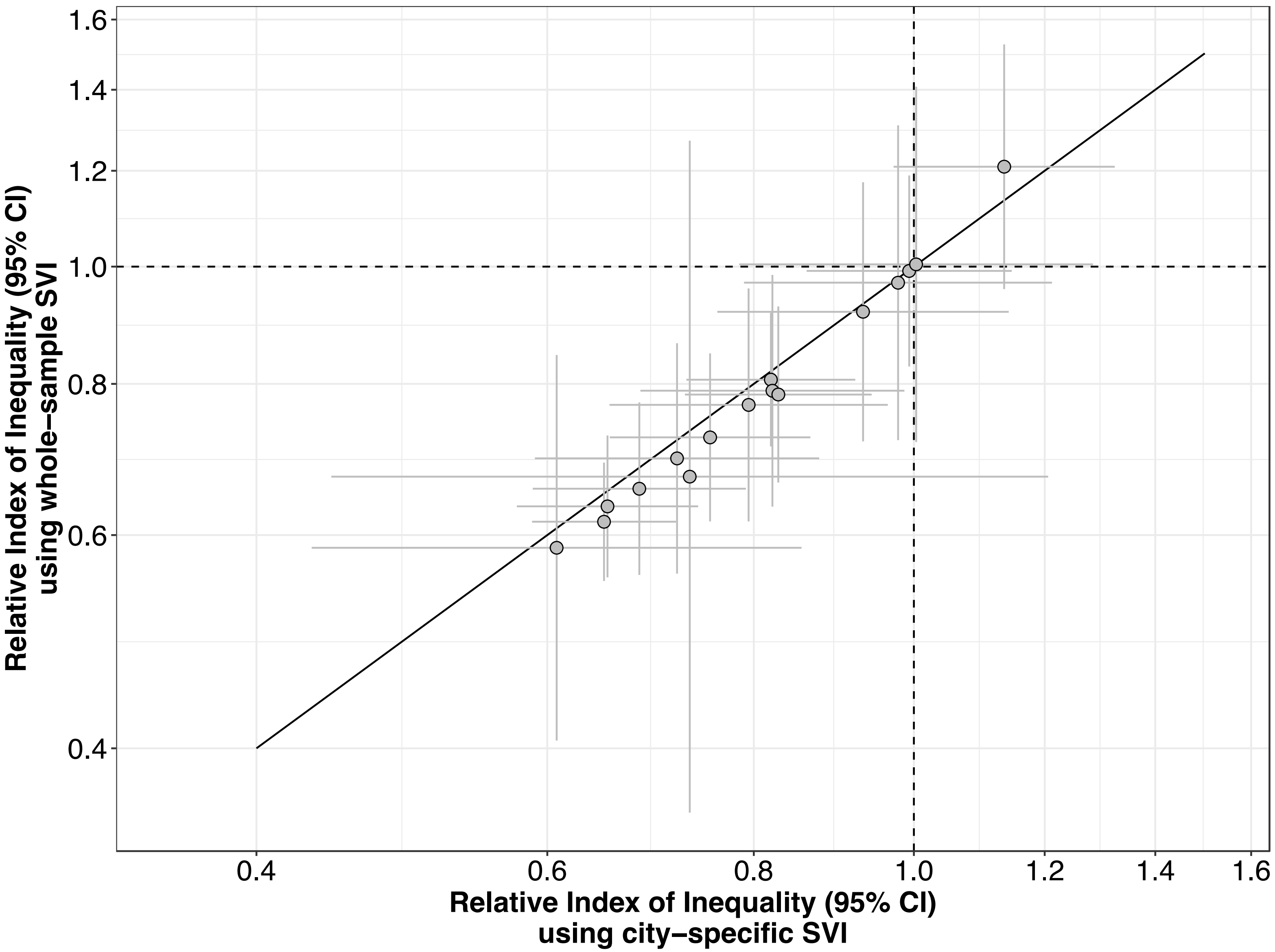

Footnote: both models are adjusted for the % of the population of the ZCTA aged 18 to 44, 45 to 64, and 65 or above. City-specific SVI is re-scaled from 0 to 1 in each city; whole sample SVI ranges from 0 to 1 in the entire sample.
