# Supplementary material for: Heterogeneity in Spatial Inequities in COVID-19 Vaccination across 16 Large US Cities
Source: Am J Epidemiol. 2022 Apr 22:kwac076. doi: 10.1093/aje/kwac076 (PMC9047229; doi:10.1093/aje/kwac076)
Supplement: Supplementary file 1 [file web_material_kwac076.docx]

**WEB MATERIAL**

**Heterogeneity in Spatial Inequities in COVID-19 Vaccination Across 16 Large US Cities**

Usama Bilal, Pricila H. Mullachery, Alina Schnake-Mahl, Heather Rollins,Edwin McCulley,

Jennifer Kolker, Sharrelle Barber, Ana V. Diez Roux

**Table of Contents**

- Web Table 1: Data Sources for this Study by city. 

- Web Table 2: fixed and random components of multilevel models for the relative and slope indices of inequality

- Web Table 3: model fit statistics and comparison of models with and without random slopes for the SVI

- Web Table 4: fixed and random components of multilevel models for the four social vulnerability index domains

- Web Figure 1: Scatterplots showing the relationship between the social vulnerability index and COVID-19 vaccination coverage in ZCTAs of 16 US cities through September 2021. 

- Web Figure 2: COVID-19 Vaccination coverage by social vulnerability quintile (whole sample) in 16 large US cities through September 2021. 

- Web Figure 3: Comparison of relative and slope index of inequalities in COVID-19 vaccination coverage by social vulnerability index in 16 large US cities through September 2021

- Web Figure 4: Comparison of age-adjusted and unadjusted relative index of inequalities in COVID-19 vaccination coverage by social vulnerability index in 16 large US cities through September 2021

- Web Figure 5: Comparison of relative indices of inequalities in COVID-19 vaccination coverage by social vulnerability index (city-standardized vs whole sample) in 16 large US cities through September 2021

**Web Table 1: Data Sources for this Study by city.** 

| **City** | **Source** | **URL** |
| --- | --- | --- |
| Long Beach, CA | California Department of Public Health | https://github.com/datadesk/california-coronavirus-data |
| Los Angeles, CA |  |  |
| Oakland, CA |  |  |
| San Diego, CA |  |  |
| San Francisco, CA |  |  |
| San Jose, CA |  |  |
| Chicago, IL | City of Chicago Department of Public Health | https://data.cityofchicago.org/ |
| Indianapolis, IN | Indiana State Department of Health | https://hub.mph.in.gov/organization/indiana-state-department-of-health |
| New York City, NY | New York Department of Health and Mental Hygiene | https://github.com/nychealth/covid-vaccine-data |
| Minneapolis, MN | Minnesota Department of Health | https://www.mn.gov/covid19/vaccine/data/index.jsp |
| Philadelphia, PA | Philadelphia Department of Public Health | https://www.opendataphilly.org/ |
| Austin, TX | Texas department of State Health Services | https://www.dshs.texas.gov/coronavirus/additionaldata/ |
| Dallas, TX |  |  |
| Fort Worth, TX |  |  |
| Houston, TX |  |  |
| San Antonio, TX |  |  |

**Footnote:** all compiled data is available at www.covid-inequities.info

**Web Table 2: fixed and random components of multilevel models for the relative and slope indices of inequality**

|  |  | **Relative Index of Inequality*** |  |  | **Slope Index of Inequality** |  |  |
| --- | --- | --- | --- | --- | --- | --- | --- |
|  |  | **Model without random slope** | **Main model** | **Main model + interaction with region** | **Model without random slope** | **Main model** | **Main model + interaction with region** |
| **Fixed** | **SVI (95% CI)** | 0.73 (0.69;0.77) | 0.75 (0.69;0.81) | 0.70 (0.64;0.76) | -17.8 (-20.5;-15.1) | -16.2 (-20.3;-12.1) | -21.1 (-25.7;-16.5) |
|  | **Age18_44** | 2.82 (2.23;3.58) | 2.65 (2.09;3.36) | 2.68 (2.12;3.40) | 74.7 (63.5;85.8) | 72.2 (61;83.5) | 73.7 (62.5;84.8) |
|  | **Age45_64** | 4.95 (2.99;8.18) | 4.61 (2.79;7.62) | 4.44 (2.69;7.33) | 115.4 (91.7;139.2) | 110.6 (86.9;134.4) | 109.7 (86.1;133.3) |
|  | **Age65+** | 4.04 (2.72;5.99) | 4.01 (2.7;5.94) | 4.26 (2.86;6.34) | 77.4 (58.7;96.1) | 77.8 (59;96.6) | 82.2 (63.5;100.9) |
|  | **South x SVI** |  |  | 1 (Ref.) |  |  | 1 (Ref.) |
|  | **Midwest x SVI** |  |  | 0.88 (0.75;1.03) |  |  | -2.5 (-10.8;5.8) |
|  | **West x SVI** |  |  | 1.27 (1.11;1.46) |  |  | 12.7 (5.6;19.8) |
|  | **Northeast x SVI** |  |  | 1.07 (0.92;1.25) |  |  | 6 (-2.4;14.4) |
| **Random** | ${\boldsymbol{τ}}_{\boldsymbol{00}}$ | 0.0133 | 0.0097 | 0.013 | 37.975 | 43.834 | 58.449 |
|  | ${\boldsymbol{τ}}_{\boldsymbol{11}}$ |  | 0.014 | 0.0011 |  | 35.425 | 9.511 |
|  | ${\boldsymbol{τ}}_{\boldsymbol{01}}$ |  | -0.0723 | -0.5624 |  | -0.339 | -1 |
|  | ${\boldsymbol{σ}}^{\boldsymbol{2}}$ | 0.046 | 0.0451 | 0.0449 | 102.506 | 100.052 | 99.653 |

**Footnote:** $τ_{00}$ corresponds to the variance of the random intercept, $τ_{11}$ corresponds to the variance of the random SVI slope, $τ_{01}$is their correlation, and $σ^{2}$  is the variance of the level 1 residuals; *RII coefficients are exponentiated

**Web Table 3: model fit statistics and comparison of models with and without random slopes for the SVI**

| **Model** | **Without Random Slope** | **With Random Slope** | **p-val of LRT*** |
| --- | --- | --- | --- |
| **RII: SVI** | logLik=77.8; df=7 | logLik=81.5; df=9 | 0.038 |
| **RII: SVI1** | logLik=89.4; df=7 | logLik=93.8; df=9 | 0.016 |
| **RII: SVI2** | logLik=80.5; df=7 | logLik=94.5; df=9 | <0.001 |
| **RII: SVI3** | logLik=33.5; df=7 | logLik=35.7; df=9 | 0.155 |
| **RII: SVI4** | logLik=35.6; df=7 | logLik=39.3; df=9 | 0.036 |
| **SII: SVI** | logLik=-3242.5; df=7 | logLik=-3238.9; df=9 | 0.046 |
| **SII: SVI1** | logLik=736.6; df=7 | logLik=739.4; df=9 | 0.097 |
| **SII: SVI2** | logLik=712.9; df=7 | logLik=729.3; df=9 | <0.001 |
| **SII: SVI3** | logLik=666.4; df=7 | logLik=674.4; df=9 | <0.001 |
| **SII: SVI4** | logLik=671.5; df=7 | logLik=680; df=9 | <0.001 |

**Footnote**: *LRT=log likelihood test testing the null hypothesis that the model with random slope has no improvement in model fit compared to the model without random slope. SVI1=socioeconomic status, SVI2=household composition and disability; SVI3=minority status and language; SVI4=housing type and transportation. df=degrees of freedom; logLik=log-likelihood

**Web Table 4: fixed and random components of multilevel models for the four social vulnerability index domains**

|  |  | **RII model*** |  |  |  | **SII model** |  |  |  |
| --- | --- | --- | --- | --- | --- | --- | --- | --- | --- |
|  |  | **SVI 1** | **SVI 2** | **SVI 3** | **SVI 4** | **SVI 1** | **SVI 2** | **SVI 3** | **SVI 4** |
| **Fixed (95% CI)** | **SVI** | 0.74 (0.69;0.8) | 0.75 (0.67;0.84) | 0.85 (0.77;0.94) | 0.85 (0.77;0.93) | -16.05 (-19.51;-12.6) | -13.72 (-19.55;-7.89) | -10.15 (-16.45;-3.84) | -11.14 (-16.63;-5.65) |
|  | **Age18_44** | 2.41 (1.9;3.06) | 1.99 (1.54;2.56) | 3.66 (2.85;4.69) | 4.88 (3.88;6.13) | 66.92 (55.6;78.25) | 60.55 (48.37;72.74) | 86.96 (75.09;98.83) | 106.11 (95.22;117) |
|  | **Age45_64** | 4.3 (2.62;7.08) | 4.33 (2.62;7.15) | 9.16 (5.48;15.31) | 9.56 (5.76;15.87) | 106.86 (83.29;130.43) | 113.47 (89.47;137.47) | 143.82 (119.25;168.39) | 150.05 (125.96;174.15) |
|  | **Age65+** | 2.86 (1.91;4.27) | 5.02 (3.44;7.33) | 4.83 (3.15;7.41) | 6.83 (4.56;10.24) | 59.69 (40.34;79.04) | 92.47 (74.34;110.59) | 84.7 (64.19;105.21) | 107.2 (87.94;126.46) |
| **Random** | ${\boldsymbol{τ}}_{\boldsymbol{00}}$ | 0.006 | 0.008 | 0.016 | 0.031 | 0.003 | 0.002 | 0.010 | 0.010 |
|  | ${\boldsymbol{τ}}_{\boldsymbol{11}}$ | 0.009 | 0.038 | 0.017 | 0.018 | 0.002 | 0.010 | 0.010 | 0.007 |
|  | ${\boldsymbol{τ}}_{\boldsymbol{01}}$ | 0.471 | -0.087 | -0.423 | -0.756 | -0.117 | -0.044 | -0.737 | -0.790 |
|  | ${\boldsymbol{σ}}^{\boldsymbol{2}}$ | 0.044 | 0.043 | 0.050 | 0.050 | 0.010 | 0.010 | 0.011 | 0.011 |

**Footnote:** $τ_{00}$ corresponds to the variance of the random intercept, $τ_{11}$ corresponds to the variance of the random SVI slope, $τ_{01}$is their correlation, and $σ^{2}$  is the variance of the level 1 residuals. *: RII fixed coefficients are exponentiated; all models are adjusted for the % of the population of the ZCTA aged 18 to 44, 45 to 64, and 65 or above; SVI1=socioeconomic status, SVI2=household composition and disability; SVI3=minority status and language; SVI4=housing type and transportation 

**Web Figure 1: Scatterplots showing the relationship between the social vulnerability index and COVID-19 vaccination coverage in ZCTAs of 16 US cities through September 2021.** 

Footnote: solid lines are lowess smoothers for each city separately. The SVI has been re-scaled for the whole sample.

**Web Figure 2: COVID-19 Vaccination coverage by social vulnerability quintile (whole sample) in 16 large US cities through September 2021.** 

**Footnote:** social vulnerability index quintiles correspond to the whole sample.

**Web Figure 3: Comparison of relative and slope index of inequalities in COVID-19 vaccination coverage by social vulnerability index in 16 large US cities through September 2021**

**Footnote:** both models adjusted for the % of the population of the ZCTA aged 18 to 44, 45 to 64, and 65 or above.

**Web Figure 4: Comparison of age-adjusted and unadjusted relative index of inequalities in COVID-19 vaccination coverage by social vulnerability index in 16 large US cities through September 2021**

**Footnote:** adjusted model controls for the % of the population of the ZCTA aged 18 to 44, 45 to 64, and 65 or above.

**Web Figure 5: Comparison of relative indices of inequalities in COVID-19 vaccination coverage by social vulnerability index (city-standardized vs whole sample) in 16 large US cities through September 2021**

**Footnote:** both models are adjusted for the % of the population of the ZCTA aged 18 to 44, 45 to 64, and 65 or above. City-specific SVI is re-scaled from 0 to 1 in each city; whole sample SVI ranges from 0 to 1 in the entire sample.
